# Depression and associated factors among Brazilian adults: the 2019 national healthcare population-based study

**DOI:** 10.1186/s12888-023-05133-9

**Published:** 2023-09-28

**Authors:** Alexandre Marcelo Hintz, Isaac Suzart Gomes-Filho, Peter Michael Loomer, Paloma de Sousa Pinho, Johelle de Santana Passos-Soares, Soraya Castro Trindade, Eneida de Moraes Marcílio Cerqueira, Claudia Maria Coêlho Alves, Yasmine Silva Santos Rios, Josicélia Estrela Tuy Batista, Ana Claudia Morais Godoy Figueiredo, Simone Seixas da Cruz

**Affiliations:** 1 Department of Health, Feira de Santana State University, Feira de Santana, Bahia Brazil; 2 School of Dentistry, University of Texas Health Science Center, San Antonio, TX USA; 3 Health Sciences Center, Federal University of Recôncavo of Bahia, Santo Antonio de Jesus, Bahia Brazil; 4 Department of Preventive Dentistry, Federal University of Bahia, Salvador, Bahia Brazil; 5 Graduate Program in Dentistry, Federal University of Maranhão, São Luis, Maranhão Brazil; 6 Epidemiology Surveillance, Federal District Health State Secretariat, Brasília, Distrito Federal Brazil

**Keywords:** Depression, Depressive disorder, Mental disorders, Associated factors, Public health

## Abstract

**Background:**

Mental disorders represent a major public health challenge worldwide, affecting 80% of people living in low- and middle-income countries. Depression, a mental disorder, is a chronic disease of long duration that causes changes in the brain, resulting from a combination of genetic, physiologic, environmental, and behavioral factors. The aim of this study was to investigate possible factors associated with depression in Brazilian adults.

**Methods:**

A population-based, cross-sectional study was carried out using the public domain database of the 2019 National Health Survey, conducted in Brazil. Depression was considered the dependent variable, and through hierarchical analysis, predictor variables were investigated such as, at the distal level—socioeconomic variables, at the intermediate level—variables related to lifestyle behavior, health condition, and history, and at the proximal level—demographic variables. Logistic regression analysis was used to obtain the adjusted Odds Ratio and the respective 95% confidence interval to identify possible factors associated with depression.

**Results:**

The study included 88,531 participant records with 10.27% diagnosed with depression. The adjusted association measurements, after selecting the independent variables in the hierarchical analysis, showed the following factors associated with depression with differing magnitudes: age, brown and white race/skin color, female sex, poor, very poor, or regular self-reported health condition, diagnosis of cardiovascular disease, work-related musculoskeletal disorder, history of smoking habit, and macroeconomic region.

**Conclusions:**

An effective strategy for preventing and managing depression in Brazilian adults must include the control of health status and lifestyle behavior factors, with actions and programs to reduce people's exposure to these factors, understanding that socioeconomic-demographic differences of each population can potentially reduce the disease burden.

## Background

Mental disorders are global public health concerns. Approximately 80% of people with mental disorders live in low- and middle-income countries [1]. The occurrence of mental disorders has increased [2] and continues to further increase, significantly changing the global profile [3, 4].

Depression is one of the most common mental disorders worldwide, with 3.8% of the population affected, that is, approximately 280 million people, including 5.0% among adults and 5.7% among adults older than 60 years [5, 6]. Depression is characterized by the presence of sad mood, apathy, lack of energy, insomnia, significant change in weight, cognitive impairment through decreased concentration and psychomotor impedance, decreased libido, feelings of guilt, negative thoughts, amongst others that can lead to suicidal thoughts [7]. It is a chronic disease of long duration, generally slowly progressive, resulting from a combination of genetic, physiologic, environmental, and behavioral factors [8].

In 2019, a population-based survey, the National Health Survey (Pesquisa Nacional de Saúde—PNS), was conducted in Brazil with the aim of also investigating chronic diseases, including diagnoses, and the use of health services and treatments [9]. The 2019 PNS findings showed that 10.2% of the Brazilian population aged 18 years and over had been diagnosed with depression, an increase of approximately 30% (7.6%) from the 2013 PNS [9]. Similar increases have been found in other studies. In 2015, the occurrence of depression in the world represented an increase of 18.4% in 10 years [10].

Depression has been considered one of the three main causes of morbidity or health problems affecting individuals’ quality of life, especially amongst women [11]. Depression causes changes in the brain affecting the availability of certain neurotransmitters, which in addition to affecting mood also decrease cognitive function and accelerate cognitive aging [12]. Mild cognitive impairment has a high risk of progression to dementia, particularly Alzheimer's disease, a mental disorder whose frequency grows exponentially with age [13]. Furthermore, depression can raise the risk for other chronic diseases such as cardiovascular disease, diabetes, and stroke, as well as for alcoholism, drug dependency, lack of productivity, relationship trouble, serious additional health issues, and premature mortality [6].

These findings point to the possible existence of factors that predispose individuals to depression that need to be identified in order to develop interventions to prevent and/or manage it [14]. To the best of our knowledge, this is the first exploratory study conducted with data from the Brazilian population, using a large population-based sample, to investigate factors potentially associated with depression in Brazilian adults using data from the 2019 PNS, using hierarchical analysis, which better identifies the factors involved in the multicausality of depression [4]. A previous descriptive study on the topic presented the changing population characteristics of depression based on two national surveys of Brazil, the 2013 and 2019 PNS, assessing sociodemographic characteristics, region of residence and health behaviors [14].

Our study adds to previous studies on the topic the knowledge of factors that can impact the occurrence of depression in the Brazilian population, contributing to the planning of prevention and control strategies for the disease. Furthermore, the findings may also contribute to the identification of probable associated factors in other populations with similar characteristics.

## Methods

This is a cross-sectional study that used the public domain database of the 2019 National Health Survey (PNS 2019), from the Brazilian Institute of Geography and Statistics (IBGE) in partnership with the Ministry of Health of Brazil. The PNS is a household survey, surveying all 27 Brazilian federative units, with the objective of expanding knowledge about the living conditions and health characteristics of the Brazilian population in order to inform public healthcare policy [9]. Currently, the PNS is conducted every five years. The 2019 PNS survey was approved by the National Research Ethics Committee of the National Health Council (Registration No. 3,529,376). All participants signed an informed consent and/or their legal guardians [14].

### Participants and sampling plan

Residents living in permanent private households in Brazil were the eligible participants of the PNS 2019. The Brazilian territory was divided into geographic Census Sectors used by the IBGE for the survey. A Census Sector is the spatial unit of information collection, established by the number of households in an area to be covered by the person who performs the data collection [9].

**Fig. 1:**
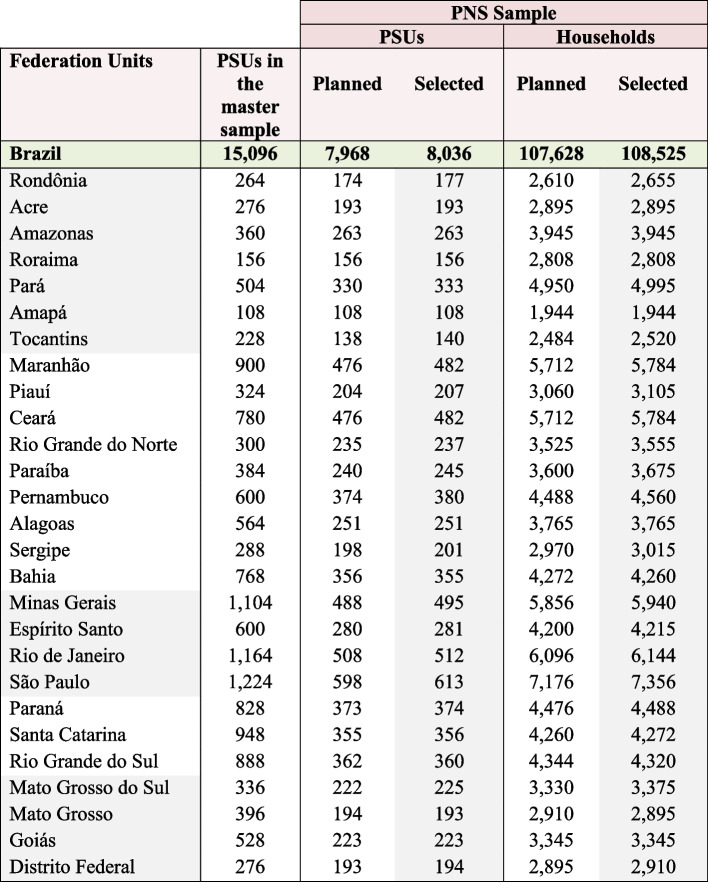
Planned and selected sizes of the sample, according to Federation Units—2019. Source: IBGE, Diretoria de Pesquisas, Coordenação de Trabalho e Rendimento (Research Directorate, Labor Coordination and Income) (IBGE, 2020). Note: PSU = Primary sampling unit. IBGE. Manual Básico da Entrevista. Pesquisa Nacional de Saúde Contínua (Basic interview manual. National Research on Continuous Health.). [Internet]. 2021. Disponível em: https://biblioteca.ibge.gov.br/visualizacao/instrumentos_de_coleta/doc5591.pdf

**Fig. 2:**
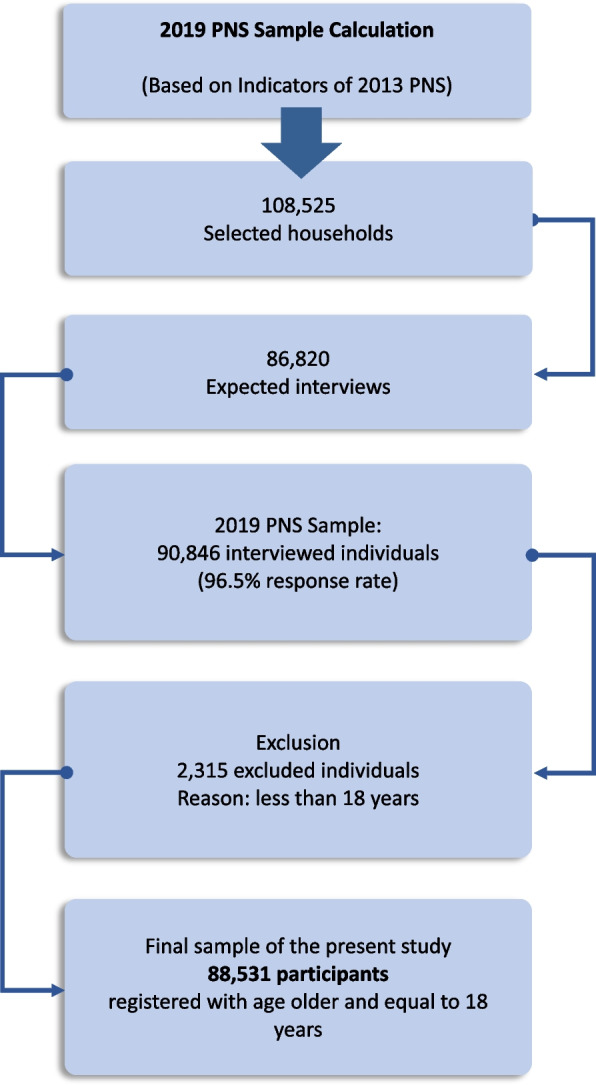
Flowchart of the 2019 PNS sample calculation and the final sample for the present study related to depression and associated factors among Brazilian adults

The PNS 2019 sample size calculation followed some specific criteria. Several indicators of interest were considered in determining the sample size of households and people. The sampling plan used was conglomerate sampling in three stages, with stratification of the primary sampling units (UPAs). In the first stage, the Census Sectors or set of sectors formed the primary sampling units; the households were the second stage units; and residents aged 15 years and over defined the third stage units [9]. More details on the sample size planned and selected for the 2019 PNS can be found in the Figs. 1 and 2.

To define the sample size of the present study, the sample of households with people, of both sexes, aged 18 years or older (selected resident) was considered [9].

### Data collection

The dependent variable considered was depression and the independent variables were those related to socioeconomic and demographic conditions, lifestyle behavior and health history.

The extraction of information of interest for the study was performed by the researchers after defining the dependent and independent variables. The selected variables came from modules C, D, I, J, N, P, Q, U, X/H and W of the 2019 PNS questionnaires. Training for data collection, including questions asked of participants through personal computer-assisted interviews, as well as measurements obtained from individuals, such as weight and height [15], are described in detail in the Health Interview Manual [16], and Stopa et al. [17].

### Dependent variable—Depression

A diagnosis of depression, the dependent variable, was made from the Patient Health Questionnaire PHQ-9, an instrument previously validated in Brazil [18], that investigated the occurrence of depressive symptoms over a two-week period prior to data collection of the epidemiological survey [17]. According to the PHQ-9 score of up to 27 points [19], depression was classified into different levels: 1 to 4 – no depression; 5 to 9 – light; 10 to 14 – moderate; 15 to 19—moderately severe; and 20 to 27—severe. According to the methodological rigor employed in the 2019 PNS, individuals who obtained a PHQ-9 score ≥ 10 points were considered as having a diagnosis of depression. Those with lower values were without depression, avoiding the "gray zone" of classification [20, 21].

### Investigated independent variables

The independent variables were identified in the PNS-2019 database, recoded, and defined as new variables for the present study and are presented in Tables 1, 2 and 3. They were subsequently distributed into distal, intermediate and proximal hierarchical levels, based on a theoretical-conceptual model of social determinants of health relating factors associated with depression [22, 23].

**Table 1:** Socioeconomic characteristics of the participants distributed according to the diagnosis of depression at the distal hierarchical level

Characteristics /Independent Variables	Depression Diagnosis Yes (%)	Depression Diagnosis No (%)	OR_crude_	95% Confidence Interval	p*
**Distal hierarchical level**					
**Macroeconomic region**					
North	5.38	94.62			
Northeast	7.43	92.57	1.41	1.29 – 1.54	< 0.01
Midwest	11.63	88.37	2.05	1.86 – 2.27	< 0.01
South	15.27	84.73	3.17	2.89 – 3.47	< 0.01
Southeast	10.47	89.53	2.31	2.12 – 2.53	< 0.01
**Level of education**					
≤ 11 years of study	9.97	90.03			
≥ 12 years of study	12.37	87.63	1.28	1.20 – 1.36	< 0.01
**Household density**					
< 4 people per household	11.05	88.95			
≥ 4 people per household	8.35	91.65	0.73	0.69 – 0.78	< 0.01
**Place of residence**					
Rural	7.68	92.32			
Urban	10.95	89.05	1.48	1.37 – 1.59	< 0.01
**Marital status**					
With partner	9.64	90.36			
Without partner	10.70	89.30	1.12	1.07 – 1.18	< 0.01
**Nature of union**					
Civil marriage	9.53	90.47			
Stable union	8.34	91.66	0.87	0.81 – 0.93	< 0.01

^*^p = *p*-value: significance level ≤ 0.05

The distal hierarchical level was composed of socioeconomic variables: macroeconomic region (north; northeast; midwest; south; southeast), level of education (≤ 11 years of study; ≥ 12 years of study), household density (< 4 people per household; ≥ 4 people per household), place of residence (rural; urban), marital status (with partner; without partner), nature of union (civil marriage; stable union).

The intermediate hierarchical level comprised those related to health condition and history, and lifestyle behavior.

Health condition and history variables were diagnosis of a chronic disease, physical or mental, or long-term illness (no; yes), limitation of usual activities due to some illness (no; yes), have a medical health plan (yes; no), have a dental plan (yes; no), time of last visit to the doctor (in years), time of last visit to the dentist (in years), hospital admission for 24 h or more in the last 12 months (no; yes), main reason for seeking care related to their own health in the last two weeks, main health care received when hospitalized (last time) in the last twelve months, general health definition (very good, good; regular; poor, very poor), considering health as a state of physical and mental well-being, and not just the absence of disease, how do you assess your state of health? (very good, good; regular; poor, very poor), Body Mass Index – BMI (≥ 18.5 kg/m^2^ to < 25 kg/m^2^—normal; < 18.5 kg/m^2^—low weight; ≥ 25 kg/m^2^ to < 29.9 kg/m^2^—overweight; ≥ 30 kg/m^2^—obesity), diagnosis of arterial hypertension (no; yes), use of medication to control high blood pressure (yes, all; yes, some; no, none), diabetes diagnosis (no; yes), use of medication to control diabetes (yes, all; yes, some; no, none), high cholesterol diagnosis (no; yes), diagnosis of cardiovascular disease (no; yes), diagnosis of arthritis or rheumatism (no; yes), diagnosis of work-related musculoskeletal disorders (no; yes), diagnosis of chronic lung disease (no; yes), diagnosis of cancer (no; yes), diagnosis of chronic kidney failure (no; yes).

Lifestyle behavior variables were practice of physical exercise or sport in the last three months (yes; no), number of hours of physical activity per week (≥ 3 h per week; < 3 h per week), current habit of smoking a tobacco product (no; yes, daily; less than daily) history of smoking habit—smoking a tobacco product daily (no; yes, daily; less than daily), self-reported assessment of oral health—teeth and gums (Very good, good; Regular, poor, very poor), eating disorder—difficulty eating because of problems with teeth or dentures (none; mild, regular, intense, very intense), number of missing upper permanent teeth (≤ 6 teeth; > 6 teeth), number of missing lower permanent teeth (≤ 6 teeth; > 6 teeth), and use of dental prosthesis (no; yes).

Finally, the proximal hierarchical level was composed of demographic variables: age (18—44 years, 45—64 years, ≥ 65 years), sex (male, female), race/skin color (black, brown, white, yellow and indigenous).

### Statistical analysis

Data analysis was performed using the statistical package STATA® version 16 (StataCorp LLC, College Station, TX, USA).

Descriptive analyzes based on the presence or absence of a diagnosis of depression were performed using simple and relative frequencies for categorical variables. The prevalence of depression was calculated. To identify factors associated with depression, groups with and without a diagnosis of depression were compared using bivariate analysis. Therefore, Pearson's chi-square test was used for categorical variables, with a significance level of 0.05. Logistic regression analysis was used, obtaining the unadjusted odds ratio and the respective 95% confidence interval (95% CI).

The hierarchical analysis of factors associated with depression was performed after the identification and selection of independent variables according to their epidemiological importance and construction of a theoretical-conceptual model on the topic [19, 20], as well as a significance level ≤ 20% obtained by bivariate analysis, using a forward strategy. Finally, at the proximal hierarchical level, variables with *p* value ≤ 0.05 were selected for the final model. The evaluation of collinearity between the independent variables was performed to better select possible factors associated, using Pearson's correlation coefficient, with the variance matrix.

The selection of independent socioeconomic variables to be included in the first level, distal hierarchical level, is related to their epidemiological importance after the construction of a theoretical model on the subject since all of them presented statistical significance. This initial decision was also taken for the inclusion of the independent variables in the other two hierarchical levels. Therefore, the variables of the distal block, macroeconomic region, education level and marital status remained associated with depression (*p* < 0.01) and were selected as adjustment variables in the next hierarchical level, the intermediate hierarchical level. In this block, together with those of the previous level, the variables related to health condition and history, and lifestyle behavior were tested: self-reported health conditions, Body Mass Index (BMI), diabetes, cardiovascular disease, Work-Related Musculoskeletal Disorder (WRMD), chronic lung disease, cancer and kidney failure, physical activity, history of smoking habit, and eating disorders. The selection of variables for the last hierarchical level was performed not only by the statistical criterion of *p* ≤ 0.20, but also by its epidemiological importance to depression. Thereby, in the last block, proximal hierarchical level, demographic variables were tested, along with those initially selected for the distal level and self-reported health condition, BMI, cardiovascular disease, WRMD, chronic lung disease, and history of smoking habit. In the proximal hierarchical level, demographic variables with *p* value ≤ 0.05 were selected for the final model.

From a theoretical-conceptual model of multicausality between independent variables and depression, as well as from the analysis of hierarchical levels, the selection of adjustment variables was performed for the multiple regression analysis. Finally, to identify factors associated with depression, logistic regression analysis was used, obtaining the crude and adjusted Odds Ratio (OR) and the respective 95% confidence interval (95% CI).

Specific procedures were used in data analysis, using weighting to correct the sample design with the definition of weights, strata and unit samples, according to the complex analysis module. The "survey" (svy) was used to perform the analysis, with consideration of the sampling design effect, non-response rates, and post-stratification weights. The analytical techniques used for weighting were the estimation of Taylor's linearized variance and the centered method to define the single sampling unit.

## Results

The final sample included 88,531 participants. Of these, 10.27% had a diagnosis of depression. The independent variables were evaluated according to the participants' depression diagnosis and are shown in Table 1—socioeconomic status, Table 2—health condition and history, and lifestyle behavior, and Table 3—demographic variables, showing the characteristics of the sample. Through logistic regression analysis and bivariate analysis, crude association measurements and the *P* value showed that most factors were associated with depression. Only the variables related to the use or not of medication to control arterial hypertension (*p* = 0.18), or to control diabetes (*p* = 0.51) and race/skin color in the yellow and indigenous category (*p* = 0.59) showed no statistically significant association. Table 4 presents a summary of the previous tables with the independent variables selected for the present study.

**Table 2:** Characteristics related to health condition and history, and lifestyle behavior of the participants distributed according to the diagnosis of depression at the intermediate hierarchical level

Characteristics /Independent Variables	Depression Diagnosis Yes (%)	Depression Diagnosis No (%)	OR_crude_	95% Confidence Interval	p*
**Intermediate hierarchical level**					
◦ Health condition and history					
**Diagnosis of a chronic disease, physical or mental, or long-term illness**					
No	0.48	99.52			
Yes	18.49	81.51	47.37	40.71 – 55.12	< 0.01
**Limitation of usual activities due to some illness**					
No	24.90	75.10			
Yes	11.20	88.80	0.38	0.36 – 0.40	< 0.01
**Have a medical health plan**					
Yes	13.00	87.00			
No	9.32	90.68	0.69	0.65 – 0.73	< 0.01
**Have a dental plan**					
Yes	11.37	88.63			
No	10.11	89.89	0.88	0.82 – 0.94	< 0.01
**Time of last visit to the doctor**					
Up to 1 year	12.02	87.98			
More than 1 year to 2 years, more than 2 years to 3 years, more than 3 years	3.24	96.76	0.25	0.22 – 0.27	< 0.01
Never went to the doctor	1.13	98.87	0.08	0.04 – 0.19	< 0.01
**Time of last visit to the dentist**					
Up to 1 year	11.37	88.63			
More than 1 year to 2 years, more than 2 years to 3 years, more than 3 years	9.47	90.53	0.81	0.78 – 0.86	< 0.01
Never went to the dentist	4.65	95.35	0.38	0.30 – 0.48	< 0.01
**Hospital admission for 24 h or more in the last 12 months**					
No	9.51	90.49			
Yes	19.04	80.96	2.24	2.09 – 2.40	< 0.01
**Main reason for seeking care related to their own health in the last two weeks**					
Prenatal consultation; Periodic medical examination; Other medical examination (admission, for driver's license, etc.); Continuation of treatment or therapy	14.92	85.08			
Mental health problem; Accident or injury; Illness or other health problem; Other	18.19	81.81	1.27	1.17 – 1.38	< 0.01
**Main health care received when hospitalized (last time) in the last twelve months**					
Complementary diagnostic tests; clinical treatment	21.36	78.64			
Psychiatric treatment; Surgery; Other; Normal birth; cesarean delivery	16.93	83.07	0.75	0.66 – 0.86	< 0.01
**General health definition**					
Very good, good	7.09	92.91			
Regular	13.99	86.01	2.13	2.02 – 2.25	< 0.01
Poor, very poor	24.65	75.35	4.29	3.97 – 4.63	< 0.01
**Considering health as a state of physical and mental well-being, and not just the absence of disease, how do you assess your state of health?**					
Very good, good	7.06	92.94			
Regular	17.05	82.95	2.71	2.56 – 2.86	< 0.01
Poor, very poor	31.18	68.82	5.97	5.49 – 6.48	< 0.01
**Body Mass Index—BMI**					
≥ 18.5 kg/m^2^ to < 25 kg/m^2^—normal	8.38	91.62			
< 18.5 kg/m^2^—low weight	12.50	87.50	1.56	0.91 – 2.67	0.10
≥ 25 kg/m^2^ to < 29.9 kg/m^2^—overweight	10.25	89.75	1.25	1.01 – 1.54	0.04
≥ 30 kg/m^2^—obesity	13.33	86.67	1.68	1.34 – 2.10	< 0.01
**Diagnosis of arterial hypertension**					
No	8.57	91.43			
Yes	15.09	84.91	1.90	1.80 – 2.00	< 0.01
**Use of medication to control high blood pressure**					
Yes, all	14.89	85.11			
Yes, some; no, none	16.20	83.80	1.11	0.96 – 1.28	0.18
**Diabetes diagnosis**					
No	10.28	89.72			
Yes	15.19	84.81	1.56	1.45 – 1.68	< 0.01
**Use of medication to control diabetes**					
Yes, all	14.89	85.11			
Yes, some; no, none	16.42	83.58	1.12	0.80 – 1.58	0.51
**High cholesterol diagnosis**					
No	9.17	90.83			
Yes	18.95	81.05	2.32	2.19 – 2.45	< 0.01
**Diagnosis of cardiovascular disease (CVD)**					
No	9.56	90.44			
Yes	22.04	77.96	2.67	2.47 – 2.89	< 0.01
**Diagnosis of arthritis or rheumatism**					
No	9.00	91.00			
Yes	24.55	75.45	3.29	3.10 – 3.51	< 0.01
**Diagnosis of Work-Related Musculoskeletal Disorders (WRMD)**					
No	9.87	90.13			
Yes	28.64	71.36	3.67	3.27 – 4.11	< 0.01
**Diagnosis of chronic lung disease**					
No	10.03	89.97			
Yes	24.97	75.03	2.98	2.60 – 3.43	< 0.01
**Diagnosis of cancer**					
No	10.04	89.96			
Yes	18.26	81.74	2.00	1.79 – 2.24	< 0.01
**Diagnosis of chronic kidney failure**					
No	10.10	89.90			
Yes	21.00	79.00	2.37	2.05 – 2.74	< 0.01
○ Lifestyle behavior					
**Practice of physical exercise or sport in the last three months**					
Yes	9.80	90.20			
No	10.59	89.41	1.09	1.04 – 1.13	< 0.01
**Number of hours of physical activity per week**					
≥ 3 h per week	9.31	90.69			
< 3 h per week	10.29	89.71	1.12	1.03 – 1.21	< 0.01
**Current habit of smoking a tobacco product**					
No	9.98	90.02			
Yes, daily; less than daily	12.21	87.79	1.25	1.17 – 1.34	< 0.01
**History of smoking habit (smoking a tobacco product daily)**					
No, I never smoked	9.11	90.89			
Yes	11.88	88.12	1.34	1.27 – 1.42	< 0.01
**Self-reported assessment of oral health (teeth and gums)**					
Very good, good	9.41	90.59			
Regular, poor, very poor	12.16	87.84	1.33	1.27 – 1.40	< 0.01
**Eating disorder (difficulty eating because of problems with teeth or dentures)**					
None	9.38	90.62			
Mild, regular, intense, very intense	16.30	83.70	1.88	1.77 – 2.00	< 0.01
**Number of missing upper permanent teeth**					
≤ 6 Teeth	10.87	89.13			
> 6 Teeth	12.11	87.89	1.13	1.04 – 1.22	< 0.01
**Number of missing lower permanent teeth**					
≤ 6 Teeth	11.15	88.85			
> 6 Teeth	12.74	87.26	1.16	1.08 – 1.26	< 0.01
**Use of dental prosthesis**					
No	9.34	90.66			
Yes	13.15	86.85	1.47	1.39 – 1.55	< 0.01

^*^p = *p*-value: significance level ≤ 0.05

**Table 3:** Demographic characteristics of the participants distributed according to the diagnosis of depression at the proximal hierarchical level

Characteristics / Independent Variables	Depression Diagnosis Yes (%)	Depression Diagnosis No (%)	OR_crude_	95% Confidence Interval	p*
**Proximal hierarchical level**					
**Age**					
18—44 years	8.09	91.91			
45—64 years	12.82	87.18	1.67	1.58 – 1.76	< 0.01
≥ 65 years	10.92	89.08	1.39	1.30 – 1.49	< 0.01
**Sex**					
Male	5.15	94.85			
Female	14.80	85.20	3.20	3.02—3.38	< 0.01
**Race/Skin color**					
Black	8.16	91.84			
Brown	8.86	91.14	1.09	1.01 – 1.19	0.05
White	12.47	87.53	1.60	1.47 – 1.75	< 0.01
Yellow and Indigenous	8.65	91.35	1.07	0.84 – 1.35	0.59

^*^p = *p*-value: significance level ≤ 0.05

**Table 4:** Unadjusted Odds Ratio of depression diagnosis according to socioeconomic-demographic variables, related to health condition and history, and lifestyle behavior of the participants

Independent Variables	Depression Diagnosis Yes (%)	Depression Diagnosis No (%)	Odds Ratio_crude_	95% Confidence Interval	p*
**Distal hierarchical level**					
**Macroeconomic region**					
Northeast	7.43	92.57	1.41	1.29 – 1.54	< 0.01
Midwest	11.63	88.37	2.05	1.86 – 2.27	< 0.01
South	15.27	84.73	3.17	2.89 – 3.47	< 0.01
Southeast	10.47	89.53	2.31	2.12 – 2.53	< 0.01
**Level of education**					
≥ 12 years of study	12.37	87.63	1.28	1.20 – 1.36	< 0.01
**Marital status**					
Without partner	10.70	89.30	1.12	1.07 – 1.18	< 0.01
**Intermediate hierarchical level**					
◦ Health condition and history					
**General health definition**					
Regular	13.99	86.01	2.13	2.02 – 2.25	< 0.01
Poor, very poor	24.65	75.35	4.29	3.97 – 4.63	< 0.01
**Body Mass Index—BMI**					
< 18.5 kg/m^2^—low weight	12.50	87.50	1.56	0.91 – 2.67	0.10
≥ 25 kg/m^2^ to < 29.9 kg/m^2^—overweight	10.25	89.75	1.25	1.01 – 1.54	0.04
≥ 30 kg/m^2^—obesity	13.33	86.67	1.68	1.34 – 2.10	< 0.01
**Diabetes diagnosis**					
Yes	15.19	84.81	1.56	1.45 – 1.68	< 0.01
**Diagnosis of cardiovascular disease (CVD)**					
Yes	22.04	77.96	2.67	2.47 – 2.89	< 0.01
**Diagnosis of Work-Related Musculoskeletal Disorders (WRMD)**					
Yes	28.64	71.36	3.67	3.27 – 4.11	< 0.01
**Diagnosis of chronic lung disease**					
Yes	24.97	75.03	2.98	2.60 – 3.43	< 0.01
**Diagnosis of cancer**					
Yes	18.26	81.74	2.00	1.79 – 2.24	< 0.01
**Diagnosis of chronic kidney failure**					
Yes	21.00	79.00	2.37	2.05 – 2.74	< 0.01
○ Lifestyle behavior					
**Number of hours of physical activity per week**					
< 3 h per week	10.29	89.71	1.12	1.03 – 1.21	< 0.01
**History of smoking habit (smoking a tobacco product daily)**					
Yes	11.88	88.12	1.34	1.27 – 1.42	< 0.01
**Eating disorder (difficulty eating because of problems with teeth or dentures)**					
Mild, regular, intense, very intense	16.30	83.70	1.88	1.77 – 2.00	< 0.01
**Proximal hierarchical level**					
**Age**					
45—64 years	12.82	87.18	1.67	1.58 – 1.76	< 0.01
≥ 65 years	10.92	89.08	1.39	1.30 – 1.49	< 0.01
**Sex**					
Female	14.80	85.20	3.20	3.02—3.38	< 0.01
**Race/Skin color**					
Brown	8.86	91.14	1.09	1.01 – 1.19	0.05
White	12.47	87.53	1.60	1.47 – 1.75	< 0.01
Yellow and Indigenous	8.65	91.35	1.07	0.84 – 1.35	0.59

^*^p = *p*-value: significance level ≤ 0.05

The hierarchical analysis provides a better selection of possible factors associated with depression (Table 5). Therefore, in the first block, distal hierarchical level, the socioeconomic variables were evaluated. In the next block, intermediate hierarchical level, variables related to health condition and history, and lifestyle behavior were tested. In the last block, proximal hierarchical level, demographic variables were tested, and those with *p* value ≤ 0.05 were selected for the final model, as previously mentioned.

**Table 5:** Hierarchical analysis of factors associated with depression. Brazil, 2019

INDEPENDENT VARIABLES	Depression	
	**OR (95% CI)**	p*
**1st. Block—DISTAL HIERARCHICAL LEVEL**		0.10^**^
* Northeast region	1.48 (1.35–1.63)	** < 0.01**
* Southeast region	2.33 (2.12–2.56)	** < 0.01**
* South region	3.19 (2.89–3.52)	** < 0.01**
* Midwest region	2.04 (1.83–2.27)	** < 0.01**
*Education level:* ≥ *12 years of study*	1.20 (1.12–1.28)	** < 0.01**
*Marital status: without partner*	1.19 (1.13–1.26)	** < 0.01**
**2nd. Block—INTERMEDIATE HIERARCHICAL LEVEL**		0.50^**^
*Self- reported health: regular*	3.18 (2.13–4.76)	** < 0.01**
*Self-reported health: poor, very poor*	4.83 (2.06–11.30)	** < 0.01**
* BMI < *18.5 kg/m*^*2*^	3.95 (1.27–12.34)	**0.02**
* BMI ≥ *25 to* ≤ *29.99 kg/m*^*2*^	1.05 (0.69–1.60)	0.81
* BMI ≥ *30 kg/m*^*2*^	1.39 (0.90–2.16)	0.14
*Diabetes: yes*	1.89 (0.46–1.73)	0.73
*Cardiovascular disease: yes*	2.43 (1.29–4.55)	** < 0.01**
*WRMD: yes*	1.88 (0.73–4.83)	0.19
*Chronic lung disease: yes*	4.65 (1.56–13.84)	** < 0.01**
*Cancer: yes*	0.85 (0.31–2.35)	0.76
*Kidney failure: yes*	1.17 (0.26–5.16)	0.84
*Practice of physical activity:* < *3 h per week*	0.90 (0.63–1.28)	0.56
*History of smoking habit: yes*	1.04 (0.69–1.56)	0.85
*Eating disorder: yes*	1.80 (0.07–3.03)	0.07
* Northeast region	1.40 (0.61–3.25)	0.43
* Southeast region	2.51 (1.11–5.67)	**0.03**
* South region	2.80 (1.21–6.50)	**0.02**
* Midwest region	2.24 (0.92–5.45)	0.07
*Education level:* ≥ *12 years of study*	1.09 (0.71–1.68)	0.70
*Marital status: without partner*	1.52 (1.05–2.21)	**0.03**
**3rd. Block—PROXIMAL HIERARCHICAL LEVEL**		0.76^**^
*Age: 45 to 64 years*	1.10 (0.86–1.40)	0.44
*Age:* ≥ *65 years*	0.70 (0.50–0.96)	**0.03**
*Sex: female*	4.08 (3.14–5.30)	** < 0.01**
*Race/skin color: brown*	1.39 (0.95–2.03)	0.09
*Race/skin color: white*	1.58 (1.07–2.34)	**0.03**
*Race/skin color: yellow and indigenous*	1.15 (0.41–3.25)	0.79
* Northeast region	1.51 (0.93–2.49)	0.10
* Southeast region	2.81 (1.73–4.55)	** < 0.01**
* South region	3.87 (2.34–6.41)	** < 0.01**
* Midwest region	3.31 (1.96–5.61)	** < 0.01**
*Education level:* ≥ *12 years of study*	1.14 (0.85–1.53)	0.37
*Marital status: without partner*	1.06 (0.84–1.33)	0.64
*Self- reported health: regular*	2.79 (2.17–3.57)	** < 0.01**
*Self-reported health: poor, very poor*	6.84 (4.61–10.16)	** < 0.01**
* BMI < *18.5 kg/m*^*2*^	1.61 (0.69–3.77)	0.27
* BMI ≥ *25 to* ≤ *29.99 kg/m*^*2*^	1.03 (0.81–1.31)	0.81
* BMI ≥ *30 kg/m*^*2*^	1.16 (0.89–1.53)	0.28
*Cardiovascular disease: yes*	2.25 (1.57–3.21)	** < 0.01**
*WRMD: yes*	2.62 (1.56–4.42)	** < 0.01**
*Chronic lung Disease: yes*	1.13 (0.53–2.41)	0.76
*History of smoking habit: yes*	1.37 (1.09–1.73)	** < 0.01**
*Eating disorder: yes*	1.34 (0.99–1.81)	0.06

^*^p = *p*-value: significance level ≤ 0.05

^**^P-value for the model's goodness-of-fit test

Finally, based on the hierarchical analysis, the independent variables selected for the final model of factors associated with depression were those with *p* ≤ 0.05. The Hosmer–Lemeshow statistical test showed good quality of the regression models and the *p* values indicated an improvement in the goodness of fit as the variables were incorporated into the analysis of the models, from distal to proximal hierarchical level.

The adjusted association measurements, obtained after selecting the independent variables in the hierarchical analysis, of the factors associated with depression are shown in Table 6. The association models for each factor were adjusted for all other independent variables, except for the main independent variable. Therefore, age was shown to be a factor associated with depression. It should be noted that in the age group 45 to 64 years, the association was positive (OR_**adjusted**_ = 1.31, 95% CI; 1.23–1.40) while among those aged ≥ 65 years the association was inverse (OR_**adjusted**_ = 0.86, 95% CI; 0.79–0.93). Race/skin color was also shown to be positively associated with depression, both in the brown and white categories. Being female had a strong positive association with depression (OR_**adjusted**_ = 3.06, 95% CI; 2.87–3.26). A self- reported health condition of “regular” (OR_**adjusted**_ = 2.65, 95% CI; 2.49–2.83), “poor or very poor” (OR_**adjusted**_ = 5.28, 95% CI; 4.77–5.84), cardiovascular disease (OR_**adjusted**_ = 1.88, 95% CI; 1.71–2.07), WRMD (OR_**adjusted**_ = 2.67, 95% CI; 2.33–3.05) and history of smoking habit were all shown to be positively associated with depression among Brazilians. The magnitude of association for self-reported health condition, cardiovascular disease and WRMD are in Table 6.

**Table 6:** Adjusted association measurements, Odds Ratio (OR), 95% confidence interval (95%CI), obtained from the final model of the hierarchical analysis of factors associated with depression. Brazil, 2019

Associated Factor	OR_adjusted_^*^ (95% IC)	p^**^
*Age: 45 – 64 years*	1.31 (1.23–1.40)	** < 0.01**
*Age:* ≥ *65 years*	0.86 (0.79–0.93)	** < 0.01**
*Sex: female*	3.06 (2.87–3.26)	** < 0.01**
*Race/skin color: brown*	1.21 (1.09–1.33)	** < 0.01**
*Race/skin color: white*	1.50 (1.36–1.66)	** < 0.01**
*Self-reported health: regular*	2.65 (2.49–2.83)	** < 0.01**
*Self-reported health: poor, very poor*	5.28 (4.77–5.84)	** < 0.01**
*Cardiovascular disease: yes*	1.88 (1.71–2.07)	** < 0.01**
*WRMD: yes*	2.67 (2.33–3.05)	** < 0.01**
*History of smoking habit: yes*	1.22 (1.15–1.30)	** < 0.01**
* Northeast region	1.19 (1.09–1.31)	** < 0.01**
* Southeast region	2.10 (1.91–2.32)	** < 0.01**
* South region	2.92 (2.62–3.24)	** < 0.01**
* Midwest region	1.93 (1.73–2.16)	** < 0.01**

The association model for each factor was fitted for all these independent variables, except the main independent variable

^*^ Adjusted for age, sex, race/skin color, self-reported health condition, diagnosis of cardiovascular disease, diagnosis of work-related musculoskeletal disorders (WRMD), history of smoking habit, and macroeconomic region

^**^p = *p*-value: significance level ≤ 0.05

*P*-value for the goodness-of-fit test of the final model: *p* = 0.30

Another factor that was positively associated with depression was the macroeconomic region (Table 6). According to its categories, there was a stronger association with depression among Brazilian residing in the South region (OR_**adjusted**_ = 2.92, 95% CI; 2.62- 3.24). The strength of the association was lower in the Southeast (OR_**adjusted**_ = 2.10, 95% CI; 1.91–2.32), Central-West (OR_**adjusted**_ = 1.93, 95% CI; 1.73–2.16) and Northeast (OR_**adjusted**_ = 1.19, 95% CI; 1.09–1.31) regions.

The Hosmer–Lemeshow statistical test verified the goodness of fit, indicating good quality of the final regression models used (Table 6).

## Discussion

The main findings of this study revealed that age, race/skin color, sex, macroeconomic region in which the individual lives, self-reported health condition, cardiovascular disease, WRMDs and history of smoking habit associated with depression in the Brazilian adult population.

The prevalence of depression in the Brazilian adult population was 10.27%, as estimated from the PNS 2019, representing approximately 16.3 million people aged 18 years and over in Brazil [15]. An occurrence of depression in the world is estimated at around 280 million people, corresponding to 3.8% of the population [5, 6]. The likely explanation for the increased prevalence in Brazil is due to the fact that most of the Brazilian population has little access to mental health services, many hours of work per day, insecurities about the future, poor quality of life and lives in a country with a high rate of violence. All these factors bring feelings of fear, worry, anxiety, and distress [24]. Worldwide, there is a trend towards depression increasing in the general population [4].

Mechanisms to explain the natural history of depression are not fully understood [25]. It is known that depression is a chronic non-communicable disease [9], resulting from a complex interaction of social, psychological, genetic, biological, physical health, environmental and behavioral factors [6, 9]. Depression causes changes in the brain that affect the availability of some neurotransmitters, which in addition to affecting mood, are also responsible for a decrease in cognitive function, accelerating “brain aging” [12].

Furthermore, systemic inflammation and vascular-disease associated risk factors are important in both the development of mild cognitive decline [26] and depression [25]. Circulating cardiovascular disease-associated risk factors, such as high serum levels of total cholesterol and triglycerides, as well as reduced high-density lipoprotein (related to increased activity of pro-inflammatory cytokines) have been shown to be associated with cognitive decline of both degenerative and vascular origin [27].

The rationale for the positive association between these disease-related factors and depression may stem from the fact that chronic metabolic insults, from different origins, may favor atherosclerosis and hyalinosis in small cerebral vessels resulting in white matter brain damage and cognitive dysfunction [26, 28], as well as depression since pro-inflammatory cytokines can signal the brain to induce a depressive state [25].

In summary, inflammation likely plays a prominent role in depression, particularly when somatic or neurovegetative symptoms are present [25]. Various health conditions, somatic disorders, and physical illnesses, which have an inflammatory basis, often occur alongside depression. Several markers of inflammatory activity are elevated in depressed compared to non-depressed individuals. Furthermore, elevations of these biomarkers appear to precede the development of depression, although bidirectional effects have also been reported [25].

Among the associated factors identified in the present study, the one with the greatest strength of positive association with depression was self-reported poor or very poor health condition. Closely behind was self-reported regular health, having a diagnosis of WRMD and cardiovascular disease [25].

Previous studies corroborate these findings, especially with regard to cardiovascular disease, which may also have a bidirectional association with depression [6, 25, 29]. Evidence from observational studies suggests that depression independently predicts incident coronary artery disease events, congestive heart failure and adverse outcomes among individuals with established cardiovascular disease [30–32].

In the present study, another factor with a strong positive association with depression was being female. The population-based survey data from both 2013 and 2019 revealed that depression was more than twice as common among Brazilian women in comparison to men [14]. Likewise, in 2014, a systematic review of the evidence on depression among Brazilian adults showed that women are more effected than men [33]. Data obtained with Brazilian students indicated that depression was 40% more frequent among female students in comparison to males [34]. The World Health Organization reiterates a higher frequency of depression among women worldwide [6].

Seeking to explain this greater occurrence, the Theory of Transduction of Social Signs of Depression describes neural, physiological, molecular and genomic mechanisms that link experiences of socio-environmental adversity with internal biological processes that drive the pathogenesis, maintenance and recurrence of depression [25]. Thereby, social stressors, such as the double jobs (home and paid) performed by women, can positively regulate inflammatory processes, inducing several depressive symptoms [25, 35]. There are also arguments based on physiological mechanisms, which point to ovarian hormonal fluctuations as responsible for women's stress, leading to inflammatory activity and reactivity at different levels, providing greater susceptibility to depression [35]. However, it is important to note that hormone expression strongly depends on the social context, that is, on several factors including age, economic status, and access to health services [35]. All these reasons help explain why women are at higher risk of developing inflammation-related depressive mood during their reproductive years, especially in those who are already at higher risk for depression.

It is important to highlight those differences in the prevalence of mental illness in men and women are well known. While mental illness is multi-factorial, the increasingly common dual role many women have, both as homecare provider while also having outside employment, likely contribute to greater prevalence in women. Therefore, it is important to consider this in the analysis of gender and mental health.

Among the variables that made up the distal hierarchical level, the only factor that was positively associated with depression in the multiple analysis was the macroeconomic region. It is worth noting that the strength of association was different for each region, being greater in those located further southern Brazil and decreasing in magnitude as the regions are in the north parts of the country. It is likely that social stressors, such as double working hours, are more prevalent in regions with greater economic development in the country.

Other variables that were associated and deserve a more careful analysis of the association measurements were age, race/skin color and history of smoking habit. Age was associated with depression, however the direction of the association varied according to age group. Among Brazilians aged 45 to 64 years, the association was positive, while among those aged 65 years and older, the association was negative. It is known that the clinical manifestation of depression in the elderly is different compared to earlier periods of life. Somatic symptoms are more prominent in late-term depression than are cognitive and emotional symptoms, compared with the disorder in young and middle adulthood [36–39]. Therefore, according to the instrument used in the PNS 2019, in which the main symptoms for the diagnosis are predominately emotional, it seems less likely that depression has been properly identified in this older age group.

The categories of brown or white race/skin color were also positively associated with depression, while the association was not statistically significant for yellow skin color and indigenous peoples. Similar findings have also found in the North American population with a higher frequency of depression in the white race [40]. On the other hand, in the Brazilian population, the results were contrary to those of the present study [41]. For the indigenous group, care should be taken with the interpretation of the result, since Brazilian sample surveys, such as the PNS, adopt as exclusion criteria, special characteristics such as sectors of indigenous villages [16]. Therefore, it cannot be considered that the indigenous population was representative in the present investigation.

History of smoking habit has been shown to be positively associated with depression. Despite intense efforts to reduce the global prevalence of smoking and the burden of attributable disease, smoking still remains a major cause of compromised health and well-being. It is known that smoking can promote deleterious changes in brain structure and neuronal function and, thereby, predispose individuals to neuropsychiatric disorders [42], such as depression. The biological mechanism that explains this relationship is mainly driven by oxidative stress, both from free radicals in smoke and from oxidative imbalance in cells. As well, inflammation caused by the effects of smoking in neuropsychiatric diseases, and other smoking-induced noncommunicable diseases, may also play a role.

A number of other depression-related factors are part of the PNS 2019 database and have been tested for their association. Many were associated when the crude measurement was estimated, but when considering the multicausality that involves depression, through adjustment techniques, most of these variables lost statistical significance.

A limitation of the present study concerns the sociocultural and economic differences typical of a country of large dimensions like Brazil, which brings together individuals from different ethnicities and social classes. Therefore, some factors investigated may have been “camouflaged”, even considering the efforts made in the elaboration of the study sample design and statistical techniques used in the multiple modeling. In addition, the design of the epidemiological survey is cross-sectional, by definition not evaluating temporality in the cause-and-effect relationship between the factors that have been shown to be associated with depression, since all variables were collected at a single point for each participant.

Another limitation is the presence of residual bias from variables that were not measured, evaluated or are unknown and therefore were not considered in the multiple analysis, such as genetic factors [43]. In addition, other variables need to be investigated in more detail in future research, such as food intake, as it has been associated with depression and food is directly associated with many variables evaluated in the present study [44].

Regarding the innovations of the study, multiple analysis performed to identify the factors associated with depression is highlighted. Initially, as the prevalence of depression was around 10%, there was no need to convert the odds ratio into a prevalence ratio [45]. The analysis also sought to evaluate the multicausality involved in depression and neutralize the effect of the other independent variables on the final association measurement. Therefore, we emphasize the benefit of employing hierarchical analysis to select the adjustment variables, through the values of the model's goodness of fit test, as more reliable method of evaluation of association.

Another strength of the study concerns the use of the instrument for the diagnosis of depression, the PHQ9, which is recognized worldwide for the definition of depressive disorder and allows good comparability of results [46].

Regarding the generalization of the findings of this study, they should be interpreted with caution, as they are for populations with similar socioeconomic and demographic characteristics, lifestyle behavior and conditions and access to health services as found in Brazil.

## Conclusions

In order to develop a truly effective strategy for the prevention and management of depression, it is necessary to understand which factors are associated and may be modifiable. These include those related to health status and lifestyle behavior, making it evident that efforts in the area of ​​health promotion should be focused here since most of these factors are largely behavioral in nature. Thus, clinical strategies to create actions and programs to reduce people's exposure to the risks of developing depression, understanding that the demographic, social and economic differences of each population can potentially reduce the disease burden.

## Data Availability

The datasets generated and/or analysed during the current study are not publicly available but are available from the corresponding author on reasonable request.

## Abbreviations

PNS: National Health Survey
IBGE: Brazilian Institute of Geography and Statistics
UPAs: Primary sampling units
PHQ-9: Patient Health Questionnaire
OR: Odds Ratio
95% CI: 95% Confidence interval
svy: "Survey"
BMI: Body Mass Index
WRMD: Work-Related Musculoskeletal Disorder
